# Protein intrinsic disorder and network connectivity. The case of 14-3-3 proteins

**DOI:** 10.3389/fgene.2014.00010

**Published:** 2014-02-03

**Authors:** Marina Uhart, Diego M. Bustos

**Affiliations:** Instituto de Investigaciones Biotecnológicas – Instituto Tecnológico de Chascomús, Universidad Nacional de San Martín – Consejo Nacional de Investigaciones Científicas y TécnicasChascomús, Argentina

**Keywords:** protein intrinsic disorder, protein interaction networks, 14-3-3 protein family, protein phosphorylation, post-translational modifications

## Abstract

The understanding of networks is a common goal of an unprecedented array of traditional disciplines. One of the protein network properties most influenced by the structural contents of its nodes is the inter-connectivity. Recent studies in which structural information was included into the topological analysis of protein networks revealed that the content of intrinsic disorder in the nodes could modulate the network topology, rewire networks, and change their inter-connectivity, which is defined by its clustering coefficient. Here, we review the role of intrinsic disorder present in the partners of the highly conserved 14-3-3 protein family on its interaction networks. The 14-3-3s are phospho-serine/threonine binding proteins that have strong influence in the regulation of metabolism and signal transduction networks. Intrinsic disorder increases the clustering coefficients, namely the inter-connectivity of the nodes within each 14-3-3 paralog networks. We also review two new ideas to measure intrinsic disorder independently of the primary sequence of proteins, a thermodynamic model and a method that uses protein structures and their solvent environment. This new methods could be useful to explain unsolved questions about versatility and fixation of intrinsic disorder through evolution. The relation between the intrinsic disorder and network topologies could be an interesting model to investigate new implicitness of the graph theory into biology.

## BRIEF INTRODUCTION TO THE 14-3-3 PROTEINS FAMILY

Phosphorylation was the first post-translational modification (PTM) described in 1954 (Burnett and Kennedy, 1954), and affects approximately one third of all proteins in the cell (Gough and Foley, 2010). Moore and Perez (1967) discovered in bovine brain extracts a family of abundant proteins, which they named “14-3-3” due to its particular elution pattern on two-dimensional DEAE-cellulose chromatography and starch gel electrophoresis. It was not until 1996 that the first biological role for the 14-3-3 proteins was elucidated: their interactions with phosphorylated partners (phospho-serine or -threonine) were a way to read this PTM in signaling cascades (Muslin et al., 1996).

This protein family is composed of ~30 kDa acidic proteins, and is ubiquitous among eukaryotic organisms. Together with kinases and phosphatases, the regulatory 14-3-3 proteins are essential components of phosphorylation-mediated signaling. The 14-3-3s have very rigid structures, and binding can induce conformational changes, not only in the binding sites of their clients (Bustos and Iglesias, 2006; Oldfield et al., 2008), but also in the entire ligand proteins (Yaffe, 2002). This might alter the stability and/or catalytic activity of the ligands (Morrison, 2009). In addition, 14-3-3 binding can hide intrinsic localization motifs, prevent molecular interactions of their partners with other proteins, and/or modulate the accessibility of a target protein to modifying enzymes such as kinases, phosphatases, or proteases (Aitken, 2006; Morrison, 2009).

Two highly conserved 14-3-3 paralogs are present yeast, seven in mammals, and up to 15 in plants (Aitken, 2006). The number of paralogs and their primary sequences are practically invariant through the mammalian phyla. The two closest paralogs are ϖ and β (86.5 % identical) and the most divergent ϖ and ε (66% identical). These differences do not affect their tridimensional structure, and specially their binding groove, in which all the paralogs contain the K49, R56, and R127 residues that are critical for 14-3-3 binding activity (Aitken, 2002).

Members of the 14-3-3 protein family self-assemble into homo- or hetero-dimers, which regulate a diverse array of cellular proteins (Morrison, 2009). It was demonstrated that the dimerization is essential for their stability and function *in vivo* (Messaritou et al., 2010). The dimeric form can be converted into monomers upon phosphorylation of the Ser58 located at the subunit interface. Monomers are less stable than dimers, and have been considered to be either less active or even inactive during binding and regulation of phosphorylated client proteins (Sluchanko and Gusev, 2012). For example, it is known that some functions of 14-3-3 proteins, like its anti-apoptotic role, are entirely dependent on the dimeric state of the protein (Woodcock et al., 2010). However, like dimers, monomers contain the phosphoserine-binding site, and therefore could – in theory – retain some functions of the dimeric 14-3-3 (Sluchanko et al., 2012). Nevertheless, further investigation about the properties of monomeric 14-3-3 is needed for understanding its yet poorly characterized role. For this reason, hereinafter in this review, each time we mention 14-3-3 without monomer or dimer specification, we will be referring to the dimeric state. Because each 14-3-3 monomer contains an independent ligand-binding channel, a dimer interacts with two motifs simultaneously, found either on a single target or on separate binding partners (Gardino et al., 2006). The latter makes the 14-3-3 dimer a scaffold protein that coordinates the physical assembly of phosphorylated components in signaling networks (Johnson et al., 2010).

The compilation of large-scale experiments together with a numerous collections of small-scale published experimental data is essential to achieve comprehensive knowledge of the interactome (De Las Rivas and Fontanillo, 2010). In an attempt to clarify the different biological roles of each 14-3-3 isoforms, a 14-3-3 protein–protein interaction network (PPIN) was built (Uhart and Bustos, 2013) by compilation of several hundreds of 14-3-3 ligands reported in low- and high-throughput studies (Pozuelo Rubio et al., 2004; Ge et al., 2010). The list of human proteins interacting with each 14-3-3 paralog was recovered from the Protein Interaction Network Analysis (PINA) platform (Wu et al., 2009), which contains a non-redundant integration of protein–protein interactions (PPI) data from the following databases: IntAct, MINT, BioGRID, DIP, HPRD, and MIPSMPact. The list was also manually revised and curated using the information of non-interacting proteins from HPRD. Our 14-3-3 full network contains 741 clients, in agreement with Mackintosh’s work (Tinti et al., 2012) that after applying an “inclusion list” and “exclusion list of common contaminants” identified 750 proteins as 14-3-3 clients. Phosphorylation, acetylation, and other PTM data were overlapped with the network to obtain a complete picture of the 14-3-3 binding partners and their modifications. The 14-3-3 network nodes are involved in diverse cellular processes like regulation of the cytoskeleton, GTPase function, membrane signaling, cell fate determination, response to insulin and TNF-alpha, cell cycle progression, and apoptosis. The 14-3-3 full network was subdivided in seven networks, each with one of the 14-3-3 mammalian paralogs as a hub (Uhart and Bustos, 2013).

## DIFFERENCES IN 14-3-3 PARALOG NETWORKS SUGGEST SPECIFIC FUNCTIONS FOR EACH 14-3-3 ISOFORM

Although originally the different isoforms of the 14-3-3 family were thought to be redundant, the fact that seven functional paralogs are strictly conserved within all mammalian species raised questions about their roles and specificity (Moreira et al., 2008; Paul et al., 2012). Yeast and human isoforms failed to reveal any isotype-specific phosphopeptide-binding activity in a full *in vitro* assay (Panni et al., 2011); however, there are many reports containing examples of *in vivo* isoform specificity (see Table 1 in Sun et al., 2009). Because of their ubiquitous conservation, it seems reasonable to think that each 14-3-3 isoform has (at least one) specific function, probably product of subfunctionalization or subneofunctionalization [a combination of neofunctionalization and subfunctionalization (He and Zhang, 2005)]. Several studies have shown tissue and/or cell cycle phase specific expression of 14-3-3 isoforms (Moreira et al., 2008). Structural data show little divergence in the phosphopeptide-binding pockets of the 14-3-3 paralogs (Yang et al., 2006), and because most 14-3-3 binding motifs conform to a few consensus sequences, it seems that isoform specificity does not reside in the binding site of the 14-3-3 partners (Uhart et al., 2011). Indeed, it most likely depends on additional contacts with the partner, probably involving residues such as anchors (Rajamani et al., 2004), outside the 14-3-3 binding motifs on the 14-3-3 partners (Uhart et al., 2011; Bier et al., 2013). Examples of these types of residues that are essential for the binding between 14-3-3 and a phosphorylated partner, other than those in the 14-3-3 binding motif, have been experimentally demonstrated to exist and characterized in the serotonin *N*-acetyltransferase (Uhart et al., 2011), and RAF1 (Bier et al., 2013). These results support the idea that phosphorylation, although necessary, is not sufficient for 14-3-3’s complex formation. Indeed, structurally constrained anchor residues play a critical function in stabilizing the PPI (Rajamani et al., 2004). This implies that specific 14-3-3 interactions could be targeted in biomedical treatments for many of the 14-3-3 related human diseases distributed world-wide (Wilker and Yaffe, 2004). As 14-3-3 proteins interact with a broad spectrum of clients within the cell, controlling specifically one of those interactions is crucial for minimizing undesired effects.

The PPINs of the different 14-3-3 isoforms, although connected through interactions with common partners, are statistically different (Uhart and Bustos, 2013); for example, Tyr is the most phosphorylated amino acid within the domains of 14-3-3ε partners. This, together with the over-representation of SH3 and Tyr_Kinase domains in 14-3-3ε PPIN, suggests that the later paralog could be particularly involved in growth factor receptors signaling pathways. In 14-3-3ϖ’s network, the number of acetylated partners (and the number of modified lysines) is significantly higher compared with each of the other isoforms PPINs. These (and other results) imply many previously unreported hidden differences of the 14-3-3 isoforms interaction networks; only a few examples have been summarized here [for the complete information, see Uhart and Bustos, 2013). These differences raised the hypothesis that subfunctionalization and/or subneofunctionalization could be possible models of how seven functional paralogs of the family were evolutionary retained. Subfunctionalization has been identified as a non-adaptive mechanism for the retention of duplicated genes in small-population species, like mammals or plants (Fernández and Lynch, 2011; Fernández et al., 2012). Proteins originated through gene duplication, as the 14-3-3 isoforms (Wenfu and Shakes, 1996), could become novel proteins through fixation of beneficial mutations (Fernández and Lynch, 2011; Fernández et al., 2012). Because the latter are generally rare, the partitioning of ancestral functions among duplicated genes by neutral evolution, or subfunctionalization, has been considered one of the primary processes for the evolution of novel proteins from duplicated genes. We do not exclude neofunctionalization as a possible evolutionary mechanism for the 14-3-3 paralogs, but because it is rare, we suggest subfunctionalization (or a combined subneofunctionalization) as more likely to have occurred in the 14-3-3 family.

To date, many examples have been demonstrated to evolve following the subfunctionalization model, which has become widely accepted in the context of duplicated gene evolution (Fernández et al., 2012). In this model, the different copies accumulate mutations that lead to specialization, such that the single-copy ancestral gene and each copy are necessary to function. The preservative role of subfunctionalization in humans and other higher eukaryotes is the result of mild mutations likely to cause a differential expression, regulation, and/or function in gene duplicates (Fernández et al., 2012), which could be the case within the 14-3-3 protein family. 14-3-3 paralogs differential expression has been documented for specific tissues, cell types, subcellular localization, and even cell cycle phase (Moreira et al., 2008). Additionally, the isoforms are differentially regulated by specific kinases (Kjarland et al., 2006), which in combination with the regulation of their expression (through time, relative amounts, and tissue, cell or subcellular localization) all contribute to fine tune their specific functions.

## INTRINSIC DISORDER IN PROTEIN–PROTEIN INTERACTION NETWORKS

Intrinsic protein disorder is a widespread phenomenon characterized by a lack of stable three-dimensional structure, and plays an important role in PPI (Uversky and Dunker, 2010). Computational predictions of protein disorder found that more than 30% of the eukaryotic proteomes are natively disordered (Ward et al., 2004). The prediction of intrinsic disorder in proteins has been revolutionary, leading to major modifications of traditional views relating protein structure and function. Extensive research has provided evidence that many proteins lack fixed structure (are disordered) under physiological conditions, and their functions depend on the unstructured rather than the structured state (Dunker et al., 2008). Disordered regions are also functionally important for protein regulation (Gsponer et al., 2008) and contain short linear motifs (short peptide sequences mainly involved in PPIs; Nguyen Ba et al., 2012). Although these regions were considered as fast evolving, a recent study found conserved sequences within them (Nguyen Ba et al., 2012). The functions of disordered regions have been classified into four categories: molecular recognition, molecular assembly, protein modification, and entropic chain activity (Dunker et al., 1998). In cellular signaling pathways, many target sites of both protein kinases and modular protein domains (such as SH3, PDZ, SH2, etc.) generally lie within disordered regions (Iakoucheva et al., 2004; Beltrao and Serrano, 2005; Fuxreiter et al., 2007; Kim et al., 2008).

In the context of PPINs, intrinsic disorder, via diversity arising from structural plasticity or flexibility, provides a means for hubs to associate with many partners (Dunker et al., 1998). This has been described as “one to many” or “many to one” signaling, depending if the disorder is in the hub or in the partners respectively. The latter is the case of the interconnected 14-3-3 isoforms networks, composed each of a 14-3-3-structured-hub interacting with many disordered binding partners (Bustos and Iglesias, 2006; Oldfield et al., 2008).

Signaling sequences and sites of PTMs are frequently, or most often, located within regions of intrinsic disorder. The flexibility of intrinsic disorder regions facilitates that a diverse array of these regions may bind to a common binding site on a single partner, as 14-3-3. Such capacity for binding diversity plays important roles in both PPINs and in gene regulation networks. Disorder-based signaling is further modulated in multicellular eukaryotes by alternative splicing, as such splicing events map to disordered regions much more often than to structured ones (Buljan et al., 2012, 2013). Associating alternative splicing with disorder could also be an explanation to the observed problems related with the folding of different length structures of isomeric amino acid sequences. The combination of disorder and alternative splicing could provide a mechanism for new signaling networks trials, enabling the evolution of cell differentiation (Buljan et al., 2013).

Finally, several small molecules of recent interest as potential drugs have been shown to act by blocking PPIs based on intrinsic disorder of one of the partners (Tompa, 2011). Studies of these examples had led to a new approach for drug discovery, and bioinformatic analysis of the human proteome suggests that various disease-associated proteins are very rich in such disorder-based drug discovery target sequences (Dunker et al., 2008).

It is widely accepted today that intrinsic disorder is an important feature in protein interactions, and contributes to build highly connected networks. An interesting classification was postulated by Gerstein and co-workers, who distinguished between singlish-interface and multi-interface hubs in the context of structural interaction networks (Kim et al., 2008). The binding partners of Singlish-interface hubs are significantly more disordered than the binding partners of multi-interface hubs, as well as more disordered than other proteins. Hence, promiscuous binding is partly mediated by disorder, but not in the interface of the singlish-interface hub itself, which is structured, rather in the interacting partners. This disordered-structured promiscuous interaction has been described in detail (Dunker et al., 2005). In accordance, a special and thus far relatively unexplored feature of intrinsically disordered proteins is that they may participate in PPIs via a novel mechanism. The current view is that most proteins in eukaryotic organisms perform their functions via transient or permanent PPIs. Structural disorder enables a special way of interactions, in which one partner utilizes a short, evolutionarily highly variable binding segment (eukaryotic linear motif) to bind a conserved cleft on the other partner (Diella et al., 2008; Davey et al., 2011). Unlike in the case of classical domain–domain interactions, this latter binding mode enables small molecules to compete with the binding, which raises the opportunity to develop potential drug target molecules that can interfere with key signal transduction pathways involved in disease states.

## INTRINSIC DISORDER IN 14-3-3 INTERACTION NETWORK

Disordered proteins are much more likely to be kinase targets compared to other proteins (Kim et al., 2008). In vein with this, interactions of 14-3-3 with their partners show characteristics, such as hydrogen bonds between side chains of 14-3-3 and the backbone of the partners and water molecules, which indicate that the partners are disordered in solution just prior to their association with 14-3-3 (Bustos, 2012). The main prerequisite of binding to 14-3-3 is the presence of phosphorylated residue(s) in a favorable (disorder promoting) molecular environment. Indeed, it is currently estimated that more than 90% of the total number of partners from all 14-3-3 mammalian isoforms do not adopt a defined three-dimensional structure, either in the entire molecule or in regions. This percentage is significantly high compared with that in other protein groups, as cell signaling and cancer-related proteins or RNA chaperons. Moreover, almost all high-affinity 14-3-3 binding motifs are contained within intrinsic disorder regions of the partners, showing the importance of disorder in 14-3-3 biology (Bustos and Iglesias, 2006).

Disordered regions can bind their targets with high specificity and low affinity (Wright and Dyson, 2009). Phosphorylation-dependent transitions from a native – disordered – state to a globular-ordered-structure may provide thermodynamic regulation of the binding. Indeed, we demonstrated that a disorder-to-order transition occurs during the binding to 14-3-3 proteins. Although this kind of transition is disfavored in terms of entropy, the formation of the complex is driven by a large enthalpy change associated with the favorable hydrogen bonding interactions involving the phosphoryl group (Bustos and Iglesias, 2006). Analysis of the crystallographic structures of 14-3-3 in complex with a variety of peptides also supports this hypothesis. Because the backbones of the peptides are highly hydrated in the bound state, this indicates that the binding peptides are likely to be unstructured before the binding (Oldfield et al., 2008). This uncouples the binding strength from specificity and renders highly specific reversible interactions, which are fundamental in cell signaling and regulation (Uhart and Bustos, 2013).

**FIGURE 1:**
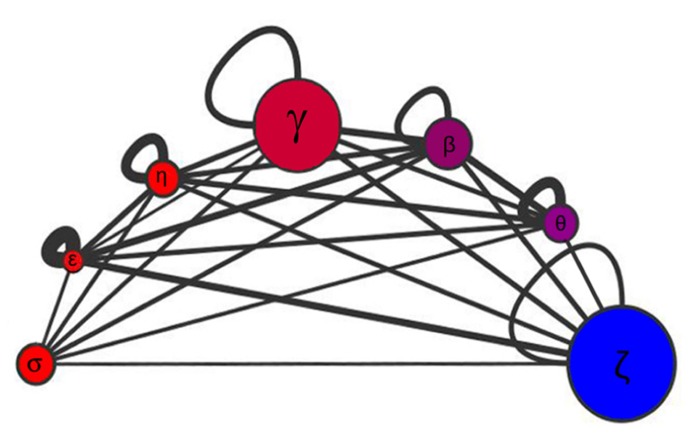
**Schematic representation of the 14-3-3 paralogs networks sizes, disorder content, similarities, and connectivity**. For each 14-3-3 paralog networks (σ, ε, η, γ, β, θ, and ζ), the size of the circle is proportional to the number of interacting partners, the disorder content is represented by color coding: red (the more disordered) to blue (the less disordered), and the clustering coefficient is proportional to the loop thickness. The thickness of the edges connecting the circles is proportional to the Jaccard similarity coefficient between networks pairs.

**FIGURE 2:**
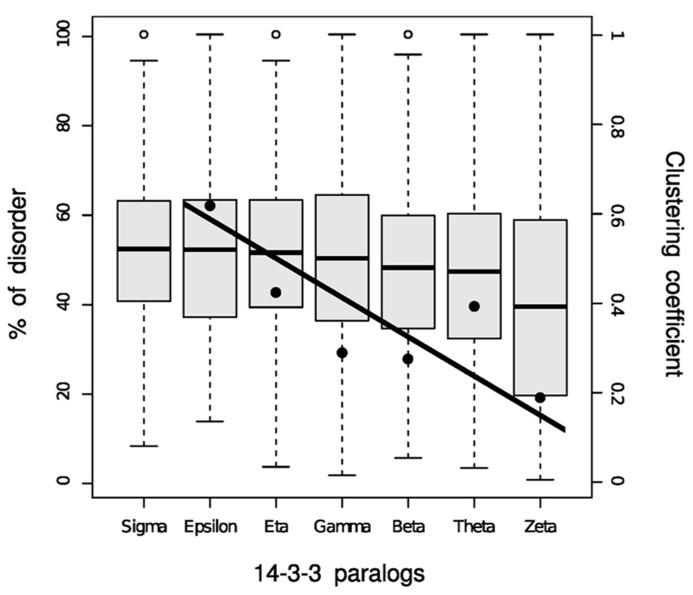
**Box plot representation of the disorder content in partners of each 14-3-3 family member**. Fisher exact test comparisons between isoforms are summarized in the Table S3 from Uhart and Bustos (2013). The clustering coefficient values (data points) for each 14-3-3 paralog network were added to the disorder graph for comparison purposes. The fitted regression line is shown (*r*^2^ = 0.715).

Disordered proteins are subject to tight regulation and targeted protein degradation, and they are substrates of twice as many kinases as are ordered proteins (Iakoucheva et al., 2004). On average, 51% of proteins substrates of kinases are highly unstructured compared with the genome wide distribution of 30% of disordered proteins. Overall, disordered regions have a much higher frequency of phosphorylation sites than ordered regions, either known or predicted. Because of 14-3-3 interactions are phosphorylation-dependent, disorder must undoubtedly play an important role in 14-3-3 PPIN. At the network level, intrinsic disorder has the function to enable proteins to interact with numerous partners. Each 14-3-3 isoform interacts with a different number of partners with a low to moderate degree of superposition (Uhart and Bustos, 2013). In **Figure 1**, the size of each circle is proportional to the number of partners in each isoform network, and the thickness of the edges connecting the circles represent the Jaccard similarity coefficient, which is a quantification of how much the networks overlap. Formally: for two samples sets, the Jaccard index is defined as the size (number of components) of their intersection divided by the size of their union. There is a low overlap between the 14-3-3 isoform-specific networks, the most similar ones being those corresponding to θ and β isoforms (Jaccard index = 0.273; as a reference, the average Jaccard index for the chaperone–protein interaction networks in *S. cerevisiae* is 0.23 (Gong et al., 2009). The 14-3-3ϖ has more partners than the other isoforms, 52% of them are exclusive. On the other side, 14-3-3ε is the isoform with the lowest number of partners with only 20 specific (for a complete list, see Table 1 in Bustos, 2012). The 14-3-3 paralog networks have different levels of disorder (**Figure 2**). This is also represented by color coding in **Figure 1**: red (the more disordered) to blue (the more ordered). Considering disordered partners as those with at least one disordered region of more than 30 consecutive amino acids (Bustos and Iglesias, 2006), 14-3-3ϖ network contains the lowest proportion of proteins with intrinsically disordered regions (73%) and 14-3-3σ network is the one with more proteins with intrinsically disordered regions, reaching up to 88%. Considering the complete 14-3-3 family, this level of disorder is probably the highest level reported so far in comparison to other protein families.

## INTRINSIC DISORDER ENHANCES THE INTER-CONNECTIVITY OF 14-3-3 PARALOG NETWORKS

The **Figure 2** shows the relationship between the clustering coefficient and the disorder content in each 14-3-3 paralog network. The clustering coefficient is a concept of the mathematical graph theory which was first defined by Watts and Strogatz (1998), and gives a notion of how connected the neighbors of a given node are. Formally, for a node *N* with *n* neighbors, there are [(*n*) × (*n* - 1)]/2 possible undirected edges between the n neighbor nodes. The clustering coefficient value is given by the actual number of edges between the neighbors n of a node *N* divided by the number of all possible edges between the nodes. The average clustering coefficient is considered the network clustering coefficient. In **Figure 1**, the clustering coefficient of each 14-3-3 isoform network is proportional to the corresponding loop thickness. Within the 14-3-3σ network, most neighbors of σ are not connected between them (thus, for most nodes, *n* = 1 because each node has a single neighbor: 14-3-3σ). This generates in the clustering coefficient calculation a division by zero, which is why is not possible to calculate the sigma clustering coefficient. With the exception of 14-3-3θ network, the percentages of intrinsic disorder in the networks correlate with their clustering coefficient (**Figures 1** and **2**). This is probably caused by the effect of the flexibility of disordered regions, which promote PPIs within each network.

**FIGURE 3:**
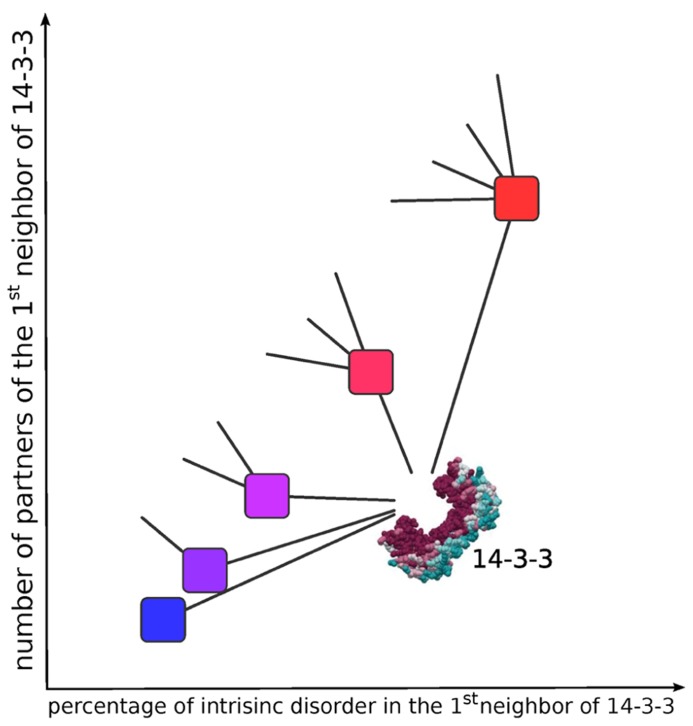
**Schematic representation of the relationship between the intrinsic disorder content and the number of neighbors in protein–protein interaction networks**. Higher intrinsic disorder content is associated to a higher number of connections (edges) of each node, resulting in higher clustering coefficients. To simplify, only one hub (14-3-3) with five hypothetical first neighbors are diagrammed. The color in the first neighbors of 14-3-3 corresponds to the degree (percentage) of intrinsic disorder content (less disordered = blue to more disordered = red). The less disordered node (blue) has only one connection (edge) to 14-3-3, and the most disordered node (red) has 5 edges, one to 14-3-3 and the other four connected to other (not diagrammed) partners.

Interactions between disordered proteins are significantly less conserved than between their ordered counterparts, displaying a higher capacity to rewire their interaction neighborhood through evolution. It has been postulated that conservation of disorder gives an evolutionary advantage, facilitating the change of interaction partners during evolution of the complexity of the entire organism interactome (Mosca et al., 2012). The **Figure 3** is a schematic representation of this principle applied to 14-3-3 PPINs.

An important issue to be discussed here is the methodology currently available to quantify protein disorder. Most algorithms used to find disordered regions in protein sequences analyze the amino acid composition in regions of 10–30 amino acids. Compared to ordered sequences, disordered ones tend to have a lower content of aromatic groups, higher net charge, higher values of the flexibility index, and greater values for hydropathy as well as other identifiable characteristics. However, this seems a tautological definition of disorder. At least two research groups are involved in programs to develop new ways to quantify protein disorder independently of the sequence composition. Camacho developed a quantitative theory (Liu et al., 2009) that makes predictions about the effect of intrinsic disorder in protein structure and function. This theory discusses analytical solutions of fundamental thermodynamic models of protein interactions in which disordered proteins are characterized by positive folding free energies (ΔG_f__>_0). Camacho’s thermodynamic model of molecular interactions can differentiate the role of disorder in binding and catalysis. In this model, folding is defined as two-state equilibrium between the unfolded state and the folded state. Molecular interactions have been traditionally described by a simple binding model that assumes that only folded proteins bind their substrates. By decoupling folding and binding, it is possible to re-define affinity, accounting for the effects of interface area, shape, hydrogen bonds, and other interactions. The formalization of this idea allows making a clear distinction between binding and folding (Liu et al., 2009). Another theoretical development was made by Fernández, who proposed that protein-water interfacial tension (PWIT) is a molecular determinant of protein interactions (Fernández and Lynch, 2011). The PWIT is generated by hydrogen bonds between particular interfacial hotspots -that are solvent accessible regions of the protein backbone- and solvent-molecules. These anomalous or atypical hydrogen bonds represent structural problems, especially with the protein folding. The inability to exclude water molecules from these interfacial hotspots causes the loss of structural integrity (Fernández and Lynch, 2011). One solution that appeared through evolution (at least in higher eukaryotes) was to bury these interfacial hotspots of accessible protein backbones through protein associations. Hence, these regions are functional indicators of PPIs. These particular hotspots have high values of intrinsic disorder propensity and *vice versa*, suggesting a relationship between these two structural properties. This opens the possibility to use the PWIT values calculation as a predictor of protein intrinsic disorder. The advantage of the later method is that PWIT is computed directly from PDB structural coordinates and not from the protein sequence, alleviating the dependence of disorder’s prediction from sequences.

These new methods could further improve our understanding of how intrinsic disorder affects 14-3-3 protein interaction networks as they may evidence previously undetected disordered regions and hidden relationships.

## CONCLUDING REMARKS

Intrinsic disorder plays a central role in the 14-3-3 protein family interaction networks, mainly shaping their topology through increasing the clustering coefficients, namely the inter-connectivity of the nodes within each network. This in turn influences practically all signal transduction pathways affected by phosphorylation, which gives an idea of how widespread the effect of intrinsic disorder is on 14-3-3 networks within a cell.

Moreover, disordered regions are now accepted as interesting areas in the structural biology field, especially after being recognized as important components in the molecular bases of PPIs. These regions differ from domain recognition sites in their shape and amino acid residue composition. The relatively new interaction and binding mode involving disordered regions differs from the classical domain-to-domain interactions in that the former could be unlocked by small molecules, opening the potential to address a number of so far “undruggable” targets.

## Conflict of Interest Statement

The authors declare that the research was conducted in the absence of any commercial or financial relationships that could be construed as a potential conflict of interest.
